# Supplementary material for: HIVprotI: an integrated web based platform for prediction and design of HIV proteins inhibitors
Source: J Cheminform. 2018 Mar 9;10:12. doi: 10.1186/s13321-018-0266-y (PMC5845081; doi:10.1186/s13321-018-0266-y)
Supplement: Supplementary file 2 — Additional file 2. Source code of HIVProtI web server. [file 13321_2018_266_MOESM2_ESM.zip › HIVprotI_Source-code/hivprotI/weka-3-6-6/doc/weka/experiment/package-summary.html]

weka.experiment


---


|  |  |  |
| --- | --- | --- |
| **Overview** \| **Package** \| Class \| **Tree** \| **Deprecated** \| **Index** \| **Help** \| **Weka's home** | |  |
| **PREV PACKAGE**   **NEXT PACKAGE** | **FRAMES**    **NO FRAMES**     **All Classes** |  |


---

## Package weka.experiment

| **Interface Summary** | |
| --- | --- |
| **Compute** | Interface to something that can accept remote connections and execute a task. |
| **RemoteExperimentListener** | Interface for classes that want to listen for updates on RemoteExperiment progress |
| **ResultListener** | Interface for objects able to listen for results obtained by a ResultProducer |
| **ResultProducer** | This interface defines the methods required for an object that produces results for different randomizations of a dataset. |
| **SplitEvaluator** | Interface to objects able to generate a fixed set of results for a particular split of a dataset. |
| **Task** | Interface to something that can be remotely executed as a task. |
| **Tester** | Interface for different kinds of Testers in the Experimenter. |

| **Class Summary** | |
| --- | --- |
| **AveragingResultProducer** | Takes the results from a ResultProducer and submits the average to the result listener. |
| **ClassifierSplitEvaluator** | A SplitEvaluator that produces results for a classification scheme on a nominal class attribute. |
| **CostSensitiveClassifierSplitEvaluator** | SplitEvaluator that produces results for a classification scheme on a nominal class attribute, including weighted misclassification costs. |
| **CrossValidationResultProducer** | Generates for each run, carries out an n-fold cross-validation, using the set SplitEvaluator to generate some results. |
| **CSVResultListener** | Takes results from a result producer and assembles them into comma separated value form. |
| **DatabaseResultListener** | Takes results from a result producer and sends them to a database. |
| **DatabaseResultProducer** | Examines a database and extracts out the results produced by the specified ResultProducer and submits them to the specified ResultListener. |
| **DatabaseUtils** | DatabaseUtils provides utility functions for accessing the experiment database. |
| **DensityBasedClustererSplitEvaluator** | A SplitEvaluator that produces results for a density based clusterer. |
| **Experiment** | Holds all the necessary configuration information for a standard type experiment. |
| **InstanceQuery** | Convert the results of a database query into instances. |
| **InstancesResultListener** | Outputs the received results in arff format to a Writer. |
| **LearningRateResultProducer** | Tells a sub-ResultProducer to reproduce the current run for varying sized subsamples of the dataset. |
| **OutputZipper** | OutputZipper writes output to either gzipped files or to a multi entry zip file. |
| **PairedCorrectedTTester** | Behaves the same as PairedTTester, only it uses the corrected resampled t-test statistic. For more information see:  Claude Nadeau, Yoshua Bengio (2001). |
| **PairedStats** | A class for storing stats on a paired comparison (t-test and correlation) |
| **PairedStatsCorrected** | A class for storing stats on a paired comparison. |
| **PairedTTester** | Calculates T-Test statistics on data stored in a set of instances. |
| **PropertyNode** | Stores information on a property of an object: the class of the object with the property; the property descriptor, and the current value. |
| **RandomSplitResultProducer** | Generates a single train/test split and calls the appropriate SplitEvaluator to generate some results. |
| **RegressionSplitEvaluator** | A SplitEvaluator that produces results for a classification scheme on a numeric class attribute. |
| **RemoteEngine** | A general purpose server for executing Task objects sent via RMI. |
| **RemoteExperiment** | Holds all the necessary configuration information for a distributed experiment. |
| **RemoteExperimentEvent** | Class encapsulating information on progress of a remote experiment |
| **RemoteExperimentSubTask** | Class to encapsulate an experiment as a task that can be executed on a remote host. |
| **ResultMatrix** | This matrix is a container for the datasets and classifier setups and their statistics. |
| **ResultMatrixCSV** | This matrix is a container for the datasets and classifier setups and their statistics. |
| **ResultMatrixGnuPlot** | This matrix is a container for the datasets and classifier setups and their statistics. |
| **ResultMatrixHTML** | This matrix is a container for the datasets and classifier setups and their statistics. |
| **ResultMatrixLatex** | This matrix is a container for the datasets and classifier setups and their statistics. |
| **ResultMatrixPlainText** | This matrix is a container for the datasets and classifier setups and their statistics. |
| **ResultMatrixSignificance** | This matrix is a container for the datasets and classifier setups and their statistics. |
| **Stats** | A class to store simple statistics |
| **TaskStatusInfo** | A class holding information for tasks being executed on RemoteEngines. |

---


|  |  |  |
| --- | --- | --- |
| **Overview** \| **Package** \| Class \| **Tree** \| **Deprecated** \| **Index** \| **Help** \| **Weka's home** | |  |
| **PREV PACKAGE**   **NEXT PACKAGE** | **FRAMES**    **NO FRAMES**     **All Classes** |  |


---
